# Social partnering alters sleep in fear-conditioned Wistar rats

**DOI:** 10.1371/journal.pone.0186017

**Published:** 2017-10-05

**Authors:** Jamie K. DaSilva, Eram Husain, Yanlin Lei, Graziella L. Mann, Adrian R. Morrison, Shanaz Tejani-Butt

**Affiliations:** 1 Department of Pharmaceutical Sciences, University of the Sciences, Philadelphia, Pennsylvania, United States of America; 2 Department of Animal Biology, University of Pennsylvania School of Veterinary Medicine, Philadelphia, Pennsylvania, United States of America; Technion Israel Institute of Technology, ISRAEL

## Abstract

Social support, when provided following a traumatic experience, is associated with a lower incidence of stress-related psychiatric disorders. Our hypothesis was that providing a social interaction period with a naive conspecific would improve sleep architecture in response to cued fear conditioning in Wistar rats. Rats were randomly assigned to either the socially isolated or socially partnered groups. Rats assigned to the socially isolated group were individually housed following electrode implantation and fear conditioning. Rats assigned to the socially partnered group were initially paired-housed, and then one rat from each pair was randomly chosen for sleep electrode implantation and fear conditioning. Rats from both groups were habituated to a recording chamber, and baseline sleep was recorded over 22 hours. One day later (Training Day), they were fear-conditioned to 10 presentations of a tone (800 Hz, 90 dB, 5 sec) co-terminating with a mild electric foot shock (1.0 mA, 0.5 sec), at 30-sec intervals. While rats in the socially isolated group were left undisturbed in their home cage for 30-min, socially partnered rats interacted for 30 minutes with their non-stressed rat partner immediately after fear conditioning and while the auditory tones were presented on Days 1 and 14. The results indicated that social interaction increased sleep efficiency in partnered rats compared to isolated rats following the fear conditioning procedure. This was due to an increase in the amount of rapid eye movement sleep (REMS) during the light phase. Evaluation of REMS microarchitecture revealed that the increase in REMS was due to an increase in the number of single REMS episodes (siREMS), which represented a more consolidated REMS pattern. A surprising finding was that partnered rats had a greater number of sequential REMS episodes (seqREMS) at Baseline, on the Training Day and on Day 1 when compared to isolated rats. The greater number of seqREMS episodes in partnered rats may be due to the partnering procedure and not fear conditioning, as the effect was also seen at Baseline. Thus it appears that while the partnering procedure may have given rise to a fragmented REMS pattern, social partnering promoted a greater consolidation of REMS in response to the fear conditioning procedure.

## Introduction

Fear is an adaptive response to threatening stimuli and functions to promote survival and maintain homeostasis [1]. Sleep alterations following a trauma can affect psychological outcome in humans [2,3]. For example, a disruption or fragmentation of rapid eye movement sleep (REMS) after a traumatic exposure may be associated with development of stress-related disorders, including post-traumatic stress disorder (PTSD) [2]. On the other hand, continuity and consolidation of REMS may facilitate the processing of emotional experiences, restore homeostasis and promote recovery [3].

The prevalence of stress-related disorders in survivors of severe trauma appears to depend on both genetic and environmental factors [4–6]. The social environment has a profound effect on the resilience or susceptibility of an individual towards developing stress-induced disorders [5, 6], and involves a complex interplay between both the quality and quantity of social interactions [7–11]. However, the impact and associated consequences of social environment on the response to trauma can vary greatly from one individual to another, and this variability is often attributed to factors such as differences in social support and coping strategies [8, 10].

Many studies have utilized animal models to investigate the mechanisms involved in susceptibility to stress disorders [12–19], but few have focused on mechanisms of resilience [3, 20, 21]. When compared to Sprague-Dawley and Long-Evans rats, the Wistar (WIS) rat strain is less sensitive to danger cues, and was found to be more mobile in a swim test when the water was soiled with urine and feces of swim-stressed rats [20]. In addition, WIS rats froze less to 2,4,5-trimethythiazoline, a commercially available, synthetic predator odor that was originally extracted from fox feces [21], suggesting that WIS rats may be a less fearful strain than other out-bred strains. A study investigating the effect of social partnering on auditory conditioned fear demonstrated that pair-housing followed by pair-exposure to the conditioned stimulus (CS) attenuated the fear-conditioned freezing response and increases in core body temperature that were observed in isolated WIS rats [22].

We have previously reported that sleep-wake behavior in the WIS rat strain, when used as a control for the stress-sensitive Wistar-Kyoto (WKY) rat strain, was unaffected by the cued fear conditioning (CFC) procedure [17]. Conversely, the WKY rat responded to CFC with a fragmented rapid eye movement sleep (REMS) pattern [17]. In a separate study, we investigated the effect of social partnering on the sleep response to CFC in the stress-sensitive WKY strain and found that socially partnered WKY rats exhibited a reduction in the REMS fragmentation that was observed in the socially isolated WKY rats [18]. In the present study, we investigated the effects of CFC on sleep, in both socially partnered and socially isolated WIS rats to understand the effect of social partnering on sleep responsiveness to CFC in a resilient strain. We hypothesized that socially partnered WIS rats would exhibit greater consolidation of REMS architecture in response to CFC when compared to socially isolated WIS rats.

## Materials and methods

### Subjects

Eight week old male WIS rats were purchased from Charles River Laboratories. Animals were randomly assigned to be individually housed (N = 6) or pair-housed (N = 6 pairs) in a temperature (22 ± 2°C) and humidity (45 ± 15%) controlled animal colony located in the University of Pennsylvania School of Veterinary Medicine. Animals were acclimated to the facility for 1-week prior to any experimental procedures. Free access to food and water was provided, except during the 10-min fear conditioning period. Animals were maintained on a 12-hr light/dark cycle, with lights on at 0700 hrs. All procedures performed on these animals were in accordance with regulations and established guidelines and were reviewed and approved by the Institutional Animal Care and Use Committee of the University of Pennsylvania.

### Social isolation and social partnering procedure

**Fig 1:**
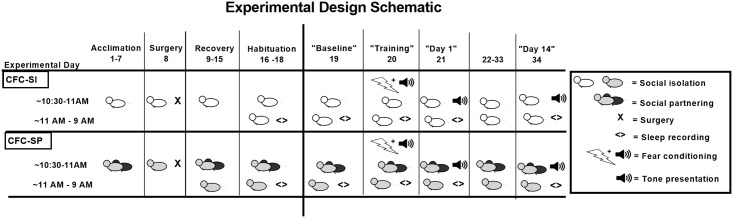
Experimental design schematic. Detailed experimental event schematic for socially isolated (top) and socially partnered (bottom) groups from acclimation through Day 14 sleep recording.

The social isolation and social partnering procedures were conducted exactly as described in [18]. Rats assigned to the socially isolated (SI) group (N = 6) were individually housed for the duration of the study. Each of the socially isolated rats were implanted with sleep recording electrodes and subjected to the fear conditioning procedure. Rats assigned to the socially partnered (SP) group (N = 12) were paired-housed for one week prior to surgery. Only one rat from each of the SP pairs was randomly chosen for electrode implantation and fear conditioning (N = 6). Rats assigned to the SP group were housed separately for the rest of the study (to maintain viability of the surgically implanted electrodes) but were provided with a daily 30-min social interaction period (10:30 AM– 11 AM) with their partner in the home cage of the surgically implanted rat. Isolated animals were left undisturbed daily for the same 30-min period (10:30 AM– 11 AM). A detailed experimental design schematic is presented in Fig 1.

### Surgical procedure

A detailed description of the surgical procedure is reported elsewhere [17, 18]. Briefly, anesthesia was induced with a mixture of ketamine (85 mg/kg) and xylazine (15 mg/kg) administered intramuscularly and was maintained via isoflurane gas (0.25%). Two pairs of EEG electrodes were implanted in contact with the dura over the frontal and sensorimotor cortices, one reference electrode was implanted over the cerebellum, and two wire EMG electrodes were attached to the neck muscles. The leads were routed to a 9-pin miniature connector and affixed onto the skull with dental acrylic. Meloxicam (0.2 mg/kg, i.m.) was administered prior to surgery and 24-h post-surgery, and gentamicin (5 mg/kg, s.c.) diluted in lactated Ringer’s solution was administered post-surgery. All rats were given a one-week recovery period. Every effort was made to minimize pain and distress, and if any animal exhibited uncommon painful responses to surgery it was immediately euthanized via anesthetic overdose.

### Signal processing

Signal processing was conducted according to [18]. Sleep recording sessions were conducted in the home cage placed inside a sound-attenuating cubicle (1m^3^). At the beginning of the session, rats were tethered to a cable counter-weighted and connected to a 12-channel, freely rotating swivel (SL6C, Plastics One). The sleep recording chamber was maintained on the same light cycle and temperature settings as the animal colony. Signals from the EEG and EMG electrodes were acquired via a Grass Model 7 polygraph amplifier system (Grass-Telefactor, USA) and Spike 2 software (Cambridge Electronics, UK). Signals were amplified (high-pass: EEG 0.3 Hz, EMG 10 Hz; low-pass: EEG 100 Hz, EMG 100 Hz) and then digitally converted by CED Power-1401 (Cambridge Electronics, UK) as used by [16–18].

### Cued fear conditioning procedure

The cued fear conditioning (CFC) procedure was conducted similarly to that of previous investigations reported by our lab [16–18]. Habituation to the tether and recording procedure was conducted over 3 days. Sleep recordings on these days were inspected for signal quality and appropriate distribution of sleep phases. Habituation data were not fully analyzed because we did not find any differences between the last day of habituation and the Baseline recording. A single 22-hr Baseline sleep recording session (~11 AM– 9 AM) took place one day prior to CFC. On the CFC Training Day, animals were fear conditioned to 10 presentations of a tone (CS: 800 Hz, 90 dB, 5 sec) and each co-terminated with a mild foot shock (US: 1.0 mA, 0.5 s) at 30-s intervals (Coulbourn Instruments Precision Shock Generator). The CFC protocol was executed in an operant chamber (Coulbourn Instruments Habitest), which was located in a designated training room that was different from the sleep recording room. Immediately following CFC, the animals were retuned to the sleep recording room where the SI rats were allowed 30-min of social isolation and the SP rats were allowed 30-min of social interaction with their designated partner rat. Sleep was recorded over 22-hr immediately following the SI and SP procedures on the Training day. Both 24-hours and again 13 days after the CFC Training procedure (Day 1 and 14, respectively), animals underwent a test recording conducted in the sleep recording room. This involved the animals being exposed to 3 tone presentations at 30-s intervals without footshock. Animals in the SI group were allowed 30-min of social isolation and animals in the SP group were allowed 30-min of social interaction while the auditory tones were presented on Days 1 and 14. Sleep was recorded over 22-hr immediately following the SI and SP procedures on Day 1 and Day 14. Immediately following the 22-hr sleep recording procedure on Day 14, animals were euthanized via rapid decapitation.

### Data analysis

Each of the 22-hr sleep records were analyzed manually using Somnologica software (Flaga hf. Medical Devices, Reykjavik, Iceland). Sleep machroarchitecture was evaluated as sleep efficiency (total sleep time/total recording time), total time (min) spent in rapid eye movement sleep (REMS), and total time spent in non-REMS (NREMS). REMS was further assessed by separating individual REMS episodes into those with an inter-REMS episode interval > 3 min, defined as single (siREMS) REMS, and those with an inter-REMS episode interval ≤ 3 min, defined as sequential (seqREMS) REMS [16–18, 23]. We then calculated the total amount of time (min) spent in siREMS and seqREMS, the total number of siREMS and seqREMS episodes and the average duration of siREMS and seqREMS episodes [14, 16–18].

### Statistical analyses

The statistical analysis was conducted using a mixed-effect model (SAS 9.1). Repeated measures with a between-subjects factor analysis of variance (ANOVA) model (between subjects factor: Environment [social partnering and social isolation]; within-subjects factor: Condition [Baseline, Training, Day 1, and Day 14]) were completed for each analysis [18]. If there was a significant main effect or interaction effect, then post hoc Tukey tests were conducted. A one-way ANOVA was conducted to determine Baseline differences between environment. All significance levels were set at *p* < 0.05.

## Results

### Sleep macroarchitecture following cued fear conditioning

**Fig 2:**
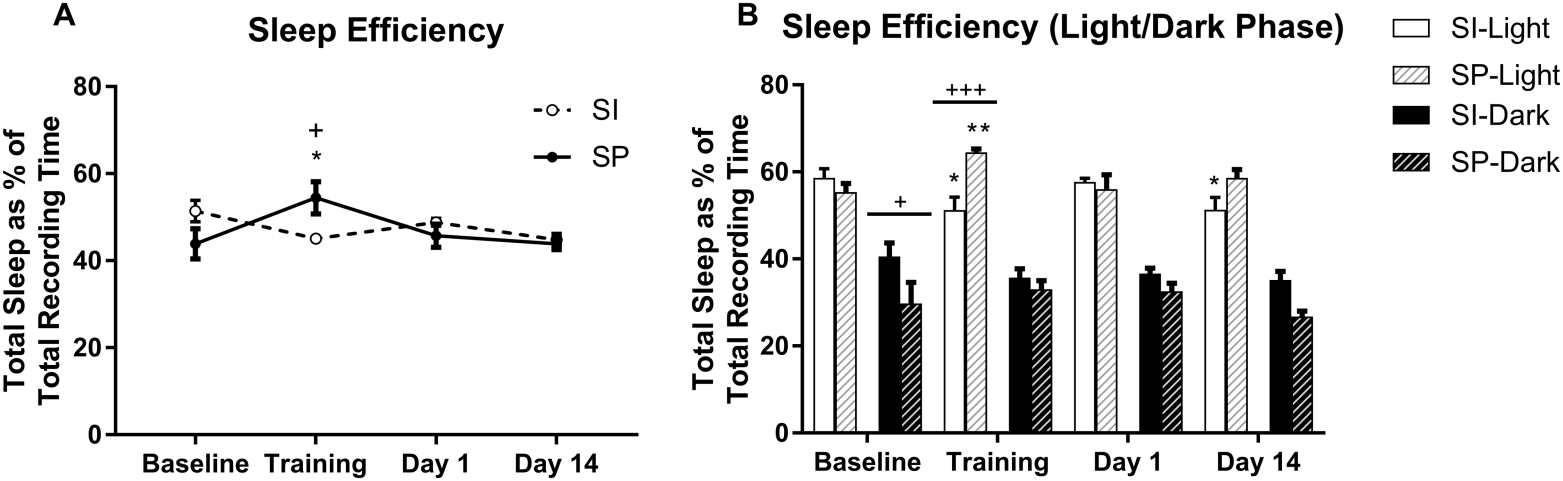
Sleep efficiency (% total sleep time/total recording time) in socially isolated (SI) and socially partnered (SP) WIS rats. A) Sleep efficiency over the 22-hr recording period. B) Sleep efficiency partitioned between the light (white bars) and dark (black bars) phase. Data are represented as mean ± S.E.M. for each of the 22-hr recording periods. Within subjects significance levels (differences relative to Baseline): Condition *P< 0.05, **P< 0.01, ***P< 0.001; and between subjects significance levels (differences between the two groups): Environment ^+^P< 0.05, ^++^P< 0.01, ^+++^P< 0.001.

Analysis of sleep efficiency over the 22-h recording period revealed a significant Condition x Environment interaction (F_3,30_ = 6.04, p<0.01). On the training day, socially partnered rats had greater sleep efficiency compared to Baseline (p = 0.05), and also when compared with socially isolated rats (p = 0.03) (Fig 2A). Separating the sleep-wake cycle into light and dark phases revealed a significant Condition x Environment interaction (F_3,30_ = 7.70, p<0.001) during the light phase, and a significant Environment effect (F_1,10_ = 6.52, p<0.05) during the dark phase. During the light phase, isolated rats had a lower sleep efficiency on the Training day (p = 0.013) and Day 14 (p = 0.014) than at Baseline, while partnered rats had greater sleep efficiency on the Training day (p = 0.003) than at Baseline. Partnered rats also had greater sleep efficiency on the Training day (p = 0.0008) than socially isolated rats. During the dark phase, partnered rats had lower sleep efficiency at Baseline (p = 0.0186) than socially isolated rats (Fig 2B).

**Fig 3:**
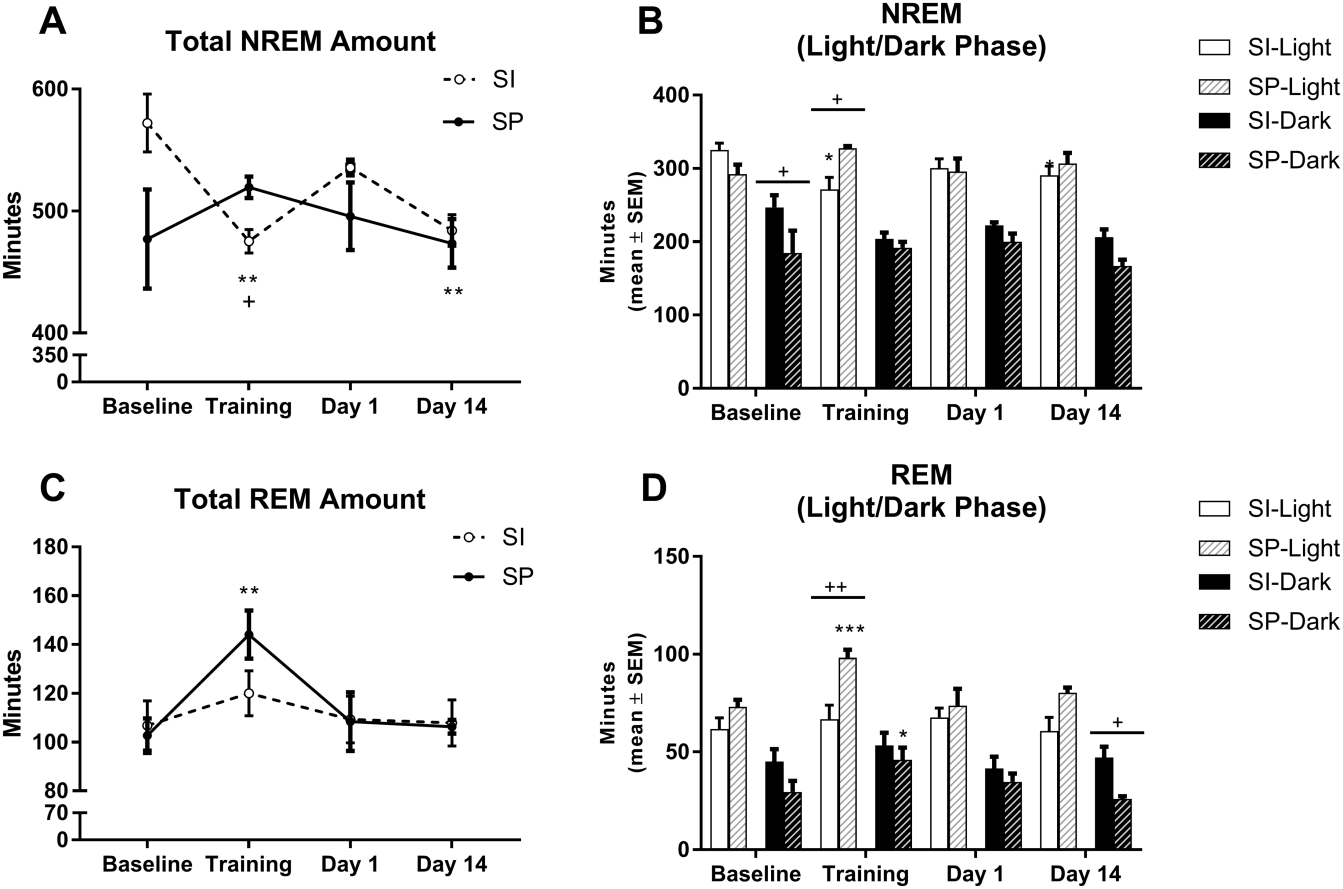
Sleep macroarchitecture in socially isolated (SI) and socially partnered (SP) WIS rats. A) Total amount (minutes) of NREMS over the 22-hr recording period. B) Total amount (minutes) of NREMS partitioned between the light (white bars) and dark (black bars) phase. C). Total amount (minutes) of REMS over the 22-hr recording period. D) Total amount (minutes) of REMS partitioned between the light (white bars) and dark (black bars) phase. Data are represented as mean ± S.E.M. for each of the 22-hr recording periods. Within subjects significance levels (differences relative to Baseline): Condition *P< 0.05, **P< 0.01, ***P< 0.001; and between subjects significance levels (differences between the two groups): Environment ^+^P< 0.05, ^++^P< 0.01, ^+++^P< 0.001.

Analysis of NREMS sleep over the 22-h recording period revealed a significant Condition x Environment interaction (F_3,30_ = 4.59, p< 0.01). While CFC decreased the amount of time spent in NREMS on the Training day (p = 0.002) and Day 14 (p = 0.004) compared with Baseline in socially isolated rats, it had no effect on socially partnered rats. However, socially partnered rats did spend more time in NREMS on Day 1(p = 0.007) when compared to their socially isolated counterparts (Fig 3A). Separating the sleep-wake cycle into light and dark phases revealed a significant Condition x Environment interaction (F_3,30_ = 4.83, p<0.01) during the light phase and a significant Environment effect (f_1,10_ = 6.55, p<0.05) during the dark phase. During the light phase, isolated rats spent less time in NREMS on the Training day (p = 0.011) than at Baseline, while partnered rats spent more time in NREMS than isolated rats on the Training day (p = 0.018). During the dark phase, partnered rats spent less time in NREMS at Baseline (p = 0.017) than isolated rats (Fig 3B).

Analysis of REMS sleep over the 22-h recording period revealed a significant Condition effect (F_3,30_ = 8.18, p< 0.01). CFC increased the amount of time spent in REMS on the Training day (p = 0.01) when compared with Baseline in socially partnered rats (Fig 3C). Separating the sleep-wake cycle into light and dark phases revealed a significant Condition effect (F_3,30_ = 5.75, p< 0.01), a significant Environment effect (F_1,10_ = 6.64, p<0.05) and a significant Condition x Environment interaction (F_3,30_ = 4.00, p<0.05) during the light phase, and a significant Condition effect (F_3,30_ = 4.69, p<0.01) during the dark phase. During the light phase, partnered rats spent more time in REMS on the Training day (p = 0.0003) than at Baseline and compared to isolated rats (p = 0.0017). During the dark phase, partnered rats spent more time in REMS on the Training day (p = 0.0198) than at Baseline, but spent less time in REMS on Day 14 (p = 0.0377) than isolated rats (Fig 3D).

### REMS microarchitecture following cued fear conditioning

**Fig 4:**
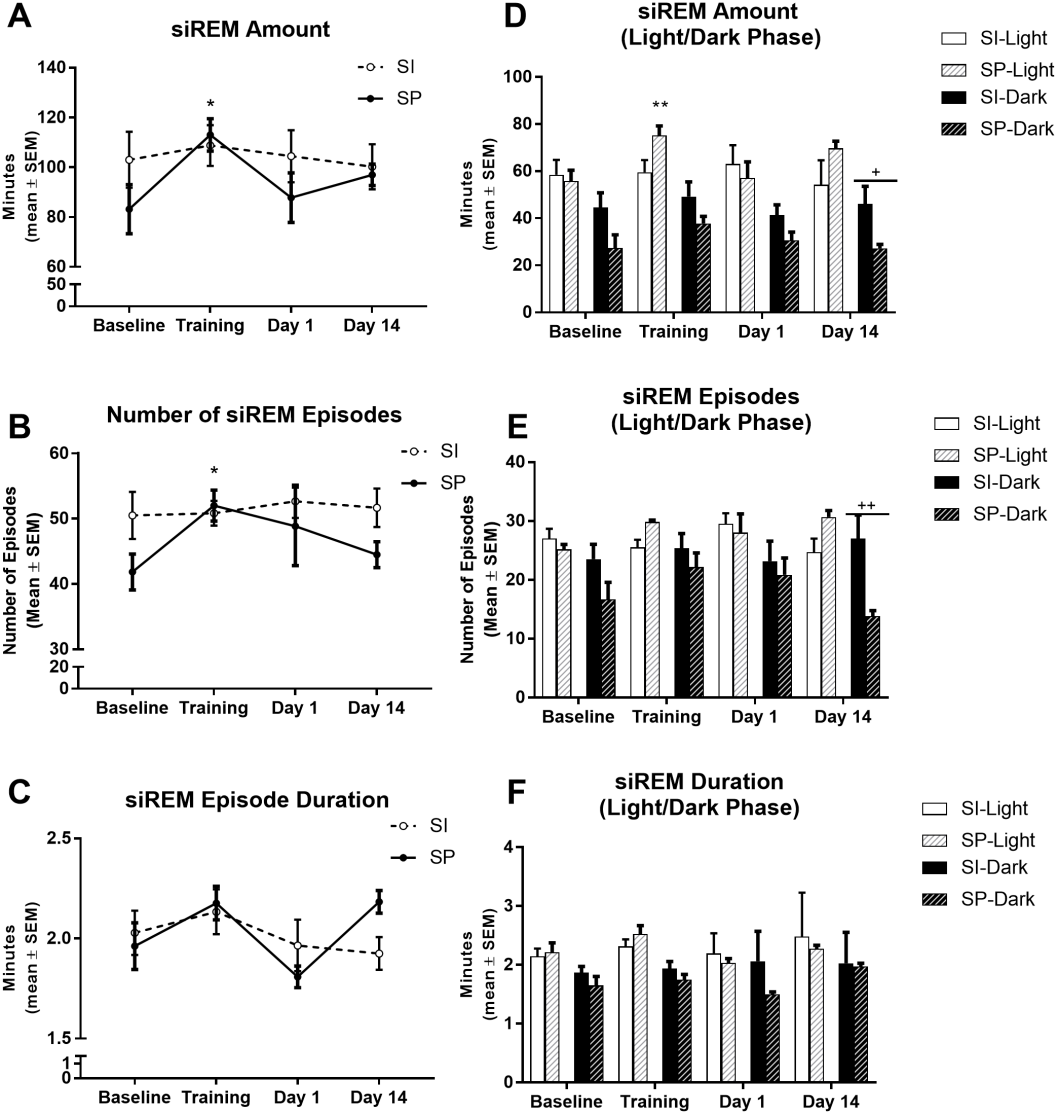
Single REMS microarchitecture in socially isolated (SI) and socially partnered (SP) WIS rats. A) Total amount (minutes) of siREMS episodes over the 22-hr recording period. B). Total number of siREMS over the 22-hr recording period. C). Mean siREMS episode duration over the 22-hr recording period. D) Total amount (minutes) of siREMS partitioned between the light (white bars) and dark (black bars) phase. E) Total number of siREMS partitioned between the light (white bars) and dark (black bars) phase. F) Mean siREMS episode duration partitioned between the light (white bars) and dark (black bars) phase. Data are represented as mean ± S.E.M. Within subjects significance levels (differences relative to Baseline): Condition *P< 0.05, **P< 0.01, ***P< 0.001; and between subjects significance levels (differences between the two groups): Environment ^+^P< 0.05, ^++^P< 0.01, ^+++^P< 0.001.

Analysis of siREMS over the 22-h recording period revealed a significant Condition effect for siREMS amount (F_3,30_ = 3.04, p< 0.05), a significant Condition x Environment interaction for number of siREMS episodes (F_3,30_ = 3.08, p< 0.05), a significant Condition effect for the duration of siREMS (F_3,30_ = 5.01, p< 0.01) and a significant Condition x Environment interaction for the duration of siREMS (F_3,30_ = 3.19, p< 0.05). In socially partnered rats, CFC increased the amount of time spent in siREMS on the Training day (p = 0.04) (Fig 4A). This increase appears to be due to an increase in the number of siREMS episodes on the Training day (p = 0.03) (Fig 4B) when compared with Baseline in socially partnered rats. Post-hoc analysis revealed that there was no significant effect on siREMS episode duration (Fig 4C).

Separating siREMS into light and dark phases revealed: a significant Condition x Environment interaction (F_3,30_ = 2.95, p< 0.05) for siREMS amount during the light phase and a significant Environment effect (F_1,10_ = 7.84, p<0.05) for siREMS amount during the dark phase; and a significant Environment effect (F_1,10_ = 5.58, p<0.05) for siREMS number during the dark phase. During the light phase, socially partnered rats spent more time in siREMS on the Training day (p = 0.0216) than at Baseline. During the dark phase, socially partnered rats spent less time in siREMS on Day 14 (p = 0.0512) (Fig 4D) and had fewer siREMS episodes (p = 0.0087) than socially isolated rats (Fig 4E). There was no effect on the duration of siREMS episodes during the light or dark phases (Fig 4F).

**Fig 5:**
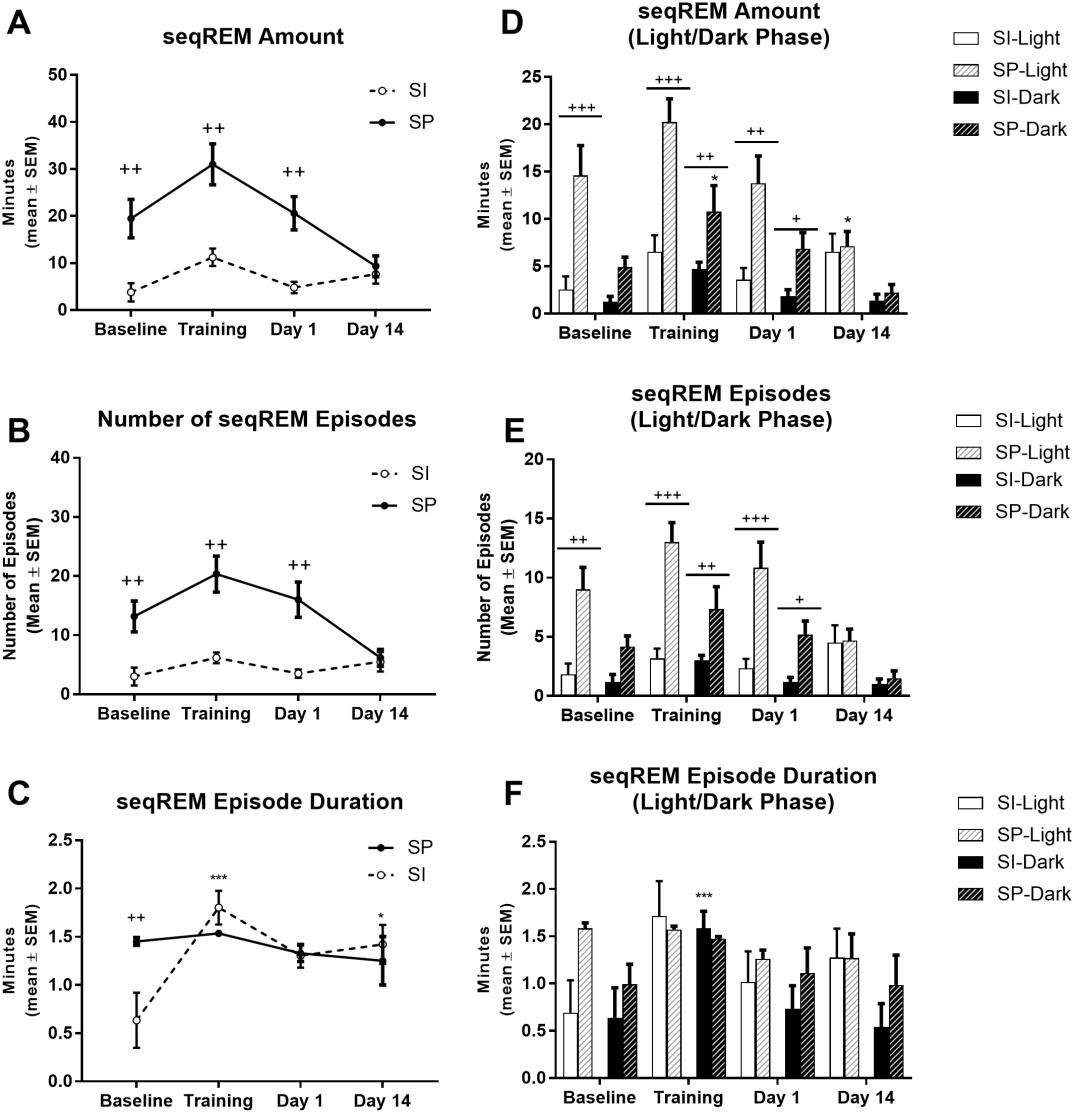
Sequential REMS microarchitecture in socially isolated (SI) and socially partnered (SP) WIS rats. A) Total amount (minutes) of seqREMS episodes over the 22-hr recording period. B). Total number of seqREMS over the 22-hr recording period. C). Mean seqREMS episode duration over the 22-hr recording period. D) Total amount (minutes) of seqREMS partitioned between the light (white bars) and dark (black bars) phase. E) Total number of seqREMS partitioned between the light (white bars) and dark (black bars) phase. F) Mean seqREMS episode duration partitioned between the light (white bars) and dark (black bars) phase. Data are represented as mean ± S.E.M. Within subjects significance levels (differences relative to Baseline): Condition *P< 0.05, **P< 0.01, ***P< 0.001; and between subjects significance levels (differences between the two groups): Environment ^+^P< 0.05, ^++^P< 0.01, ^+++^P< 0.001.

Analysis of seqREMS amount over the 22-h recording period revealed a significant Environment effect for seqREMS amount (F_1,10_ = 28.06, p< 0.001), a significant Environment effect for the number of seqREMS episodes (F_1,10_ = 29.21, p< 0.001), a significant Condition effect for the duration of seqREMS (F_3,30_ = 3.65, p< 0.05) and a significant Condition x Environment interaction for the duration of seqREMS (F_3,30_ = 3.37, p< 0.05). Socially partnered rats spent more time in seqREMS at Baseline (p = 0.006), on the Training day (p = 0.002) and on Day 1 (p = 0.002) compared to isolated rats (Fig 5A). The greater amount of time spent in seqREMS in partnered rats appears to be due to a greater number of seqREMS episodes at Baseline (p = 0.007), on the Training day (p = 0.001) and on Day 1 (p = 0.002) when compared with isolated rats (Fig 5B). Socially partnered rats had a greater seqREMs episode duration at Baseline (p = 0.0076) when compared with isolated rats. Whereas in isolated rats, CFC increased the duration of seqREMS episodes on the Training day (p = 0.0004) and Day 14 (p = 0.0193) compared to Baseline (Fig 5C).

Separating seqREMS into light and dark phases revealed: a significant Condition effect (F_3,30_ = 4.00, p<0.05), Environment effect (F_1,10_ = 21.53, p<0.001) and Condition x Environment interaction (F_3,30_ = 4.78, p<0.01) for seqREMS amount during the light phase, and a significant Condition effect (F_3,30_ = 8.72, p<0.001) and Environment effect (F_1,10_ = 11.98, p<0.01) for seqREMS amount during the dark phase; a significant Environment effect (F_1,10_ = 24.09, p<0.001) and Condition x Environment interaction (F_3,30_ = 5.85, p<0.01) for seqREMS number during the light phase, and a significant Condition effect (F_3,30_ = 5.38, p<0.01), and Environment effect (F_1,10_ = 17.49, p<0.01) for seqREMS number during the dark phase; a significant Condition effect (F_3,30_ = 4.52, p<0.01) for seqREMS duration during the dark phase. During the light phase, partnered rats spent less time in seqREMS during the light phase on Day 14 (p = 0.0145) than at Baseline (Fig 5D). Partnered rats also spent more time in seqREMS at Baseline (p = 0.0006), on the Training day (p = 0.0004) and Day 1 (p = 0.0087) than isolated rats (Fig 5D). This difference appears to be due to partnered rats having a greater number of seqREMS episodes at Baseline (p = 0.0041), on the Training day (p<0.0001) and Day 1 (p = 0.0006) than isolated rats during the light phase (Fig 5E). During the dark phase, partnered rats spent more time in seqREMS on the Training day (0.0014) than at Baseline (Fig 5D). Partnered rats also spent more time in seqREMS during the dark phase on the Training day (p = 0.0096) and Day 1 (p = 0.0278) than isolated rats (Fig 5D). This difference appears to be due to partnered rats having a greater number of seqREMS episodes on the training day (p = 0.0057) and Day 1 (p = 0.0410) than isolated rats during the dark phase (Fig 5E). During the dark phase, isolated rats had shorter duration of seqREMS episodes at Baseline than on the Training day (p = 0.0214) (Fig 5F). This appears to be due to a number of isolated rats not having any seqREMS at Baseline, thus lowering the group mean overall (see S4 File).

## Discussion

This report characterizes the sleep macro and micro-architecture in socially isolated and partnered WIS rats immediately following CFC (Training Day) and after re-exposure to the auditory conditioned stimulus on Days 1 and 14. Although rats have polyphasic sleep distributed over short periods [24, 25], this study partitioned sleep parameters between the light/dark phases to better understand the distribution of sleep architecture. This was done to identify long-term, persistent changes in sleep architecture over 8–12 hour intervals up to fourteen days following CFC. The results indicated that, immediately following the fear conditioning procedure, socially partnered rats exhibited increased sleep efficiency during the light phase. This increased sleep efficiency could be due to an increase in the amount of time spent in REMS during the light phase. Socially partnered rats also spent more time in NREMS during the light phase following fear conditioning on the training day compared to their socially isolated counterparts. Conversely, socially isolated rats responded to fear conditioning with a decreased amount of NREMS. Analysis of REMS microarchitecture revealed that the CFC-induced increase in REMS in socially partnered rats was due to an increase in the amount of siREMS during the light phase as well as the number of siREMS episodes over the entire 22-hr recording period, suggesting a more consolidated REMS response to fear conditioning. A surprising finding in this study was that socially partnered rats had an overall greater amount of seqREMS episodes when compared to socially isolated rats during both the light and dark phase.

Social interactions have been reported to increase sleep efficiency in humans [26–30]. In agreement, the present study found that sleep efficiency and the amount of REMS were increased in socially partnered rats during the light phase following fear conditioning on the Training day. Multiple studies have reported that an increase in sleep, specifically REMS, is an important adaptive behavior that promotes recovery from a stressful situation [3, 5, 31–34]. [33] reported that immobilization stress lasting longer than 4 hours suppressed the sleep rebound that was otherwise observed with shorter duration stress periods. Furthermore, it has been reported that the suppression of the sleep rebound was mediated by prolonged increases in corticosterone [33]. Therefore, social partnering may be facilitating a physiological homeostatic response to fear conditioning, in the form of a stress-induced sleep rebound [30]; functioning as an adaptive response to promote recovery from the fear conditioning procedure itself. In socially isolated rats, the CFC-induced decrease in NREMS immediately following training is consistent with other reports of CFC-induced changes in WIS rats [35]. For example, [35] investigated the sleep response to extinction of contextual fear conditioning in WIS rats, and reported that fear conditioning suppressed NREMS immediately following shock training, whereas the fear extinction procedure increased REMS during subsequent sleep.

When REMS was separated into single and sequential episodes [23], we previously reported that CFC did not affect REMS microarchitecture in socially isolated WIS rats over a 4-h sleep recording either 24 hours or 14 days post-CFC [17]. In agreement with [17], the present study suggests that CFC had no effect on REMS microarchitecture in socially isolated WIS rats on day 1 or Day 14. However, fear conditioning did increase the amount and number of siREMS episodes in socially partnered rats immediately following the fear conditioning procedure during the light phase on the Training day. It has been proposed that siREMS episodes represent consolidated REMS, whereas seqREMS episodes represent a fragmented REMS pattern [17, 23]. Representative hypnograms of this characteristic REMS microarchitecture in WIS rats have been published in [17]. We have also reported that social partnering promotes consolidation (via increased siREMS) of the fragmented REMS pattern induced by CFC in the stress-sensitive WKY rat strain [18]. Taken together with the current results, our data indicate that social partnering promotes a consolidated REMS response to the fear conditioning procedure via an increase in the number of siREMS episodes regardless of a resilient or stress-sensitive phenotype.

Consistent with our previous work [17], the present study indicates that CFC did not affect seqREMS amount in socially isolated WIS rats. Isolated rats had a lower average seqREMS episode duration than partnered rats at Baseline. This could be due to a number of isolated rats not having any seqREMS, thus lowering the group mean overall (see S4 File). A surprising finding in this study was that socially partnered rats had a greater amount of seqREMS than socially isolated rats at Baseline, on the Training day and on Day 1. The presence of the effect at Baseline suggests that the greater amount of seqREMS may be due to the partnering procedure and not a result of fear conditioning. The lack of an effect on seqREMS on Day 14 may indicate habituation to the partnering procedure. While other reports investigating stress in a social environment utilized continuous pair-housing paradigm [22], the social partnering procedure used in this study required separation of the rat pairs after 30 minutes of interaction each day. This was necessary to protect the viability of the sleep recording electrodes.

Given that the primary goal was to evaluate the effect of fear conditioning on sleep in socially partnered rats, it was not possible to determine whether the greater number of seqREMS in partnered rats was due to the methodological limitation of separating the rat pairs, or the complex transfer of social cues between rat pairs. While studies have investigated the effects of separation on wake behavior [36–39], little is known regarding the sleep response when provided the opportunity to interact with, and then be separated from, a familiar conspecific. [40] reported that naïve, outbred, group housed mice have a REMS profile that include a greater number of short duration episodes when compared to single housed mice, which is similar to the characteristic short duration seqREMS episodes previously described [17, 23]. Taken together, this suggests that group housing alone may be sufficient to induce short duration REMS episodes that may be similar to a seqREMS pattern. However, in [18] we had previously hypothesized that the reduction in seqREMS observed in socially partnered WKY rats could be due to mechanisms by which social support suppresses heightened activity of the hypothalamo-pituitary-adrenal (HPA) axis [8, 41–44]. In contrast, postnatal maternal separation results in hyper-responsiveness of the HPA axis to aversive stimuli in adult rodents [37]. Furthermore, [39] reported that rodents separated from a familiar partner demonstrated increased levels of corticosterone [39]. Hyper-responsiveness of the HPA axis following separation of the WIS rat pairs could potentially disrupt the maintenance of REMS; contributing to the enhancement of seqREMS. Thus, it is possible that even though social partnering promoted the consolidation of REMS in response to the fear conditioning procedure in WIS rats, the social partnering procedure alone may have induced a more fragmented REMS pattern, albeit the mechanism of this phenomenon remains unclear. Further investigation of the effect of social partnering alone and following fear conditioning would be complimented by measuring adrenocorticotropic hormone (ACTH) and corticosterone levels to fully characterize the influence of stress and HPA responsiveness on changes in sleep parameters [32–34].

## Conclusions

Socially partnered WIS rats showed increased sleep efficiency and responded to CFC with an increased number of siREMS episodes. The overall greater number of seqREMS episodes in socially partnered rats compared to their socially isolated counterparts appears to be driven by the social partnering procedure itself. The objective and subjective aspects of social relationships are varied and complex, making it a difficult dynamic to study in a rodent model. While social partnering appeared to promote the consolidation of REMS in response to the fear conditioning procedure, the necessary separation of the rat pairs may have uncovered an important finding that WIS rats respond to separation from their partners with a fragmented REMS pattern. This is especially interesting when we consider evidence that the WIS rat strain is more resilient than other rat strains [17, 20, 21]. While social support has been proven to be beneficial [6, 29, 30, 45, 46], the quantity and quality of social support required to have a beneficial effect is unknown [11, 47]. In fact, growing evidence suggests that it is the quality of the social support structure and the perception of ‘social connectedness’ that is predictive of psychological outcomes [48–50]. Further studies are required to understand the mechanisms by which the social partnering procedure may have caused this enhancement of seqREMS in the resilient WIS rat strain.

## Supporting information

S1 File Individual animal data for Fig 2. (XLSX)

S2 File Individual animal data for Fig 3. (XLSX)

S3 File Individual animal data for Fig 4. (XLSX)

S4 File Individual animal data for Fig 5. (XLSX)
